# Research on temperature detection method of liquor distilling pot feeding operation based on a compressed algorithm

**DOI:** 10.1038/s41598-024-64289-w

**Published:** 2024-06-10

**Authors:** Xiaolian LIU, Shaopeng Gong, Xiangxu Hua, Taotao Chen, Chunjiang Zhao

**Affiliations:** 1 School of Mechanical Engineering, Taiyuan University of Science and Technology, Taiyuan, 030024 China; 2 College of Intelligent Manufacturing Industry, Shanxi Electronic Science and Technology Institute, Linfen, 041000 China

**Keywords:** Lightweight model, YOLO v5n, Knowledge Distillation, Model Pruning, Electrical and electronic engineering, Computer science

## Abstract

In the process of feeding the distilling bucket after vapor detection, the existing methods can only realize the lag detection after the steam overflow, and can not accurately detect the location of the steam, etc. At the same time, in order to effectively reduce the occupancy of the computational resources and improve the deployment performance, this study established infrared image dataset of fermented grains surface, and fused the YOLO v5n and the knowledge distillation and the model pruning algorithms, and an lightweight method YOLO v5ns-DP was proposed as as a model for detecting temperature changes in the surface layer of fermented grains during the process of feeding the distilling. The experimental results indicated that the improvement makes YOLOv5n improve its performance in all aspects. The number of parameters, GLOPs and model size of YOLO v5ns-DP have been reduced by 28.6%, 16.5%, and 26.4%, respectively, and the mAP has been improved by 0.6. Therefore, the algorithm is able to predict in advance and accurately detect the location of the liquor vapor, which effectively improves the precision and speed of the detection of the temperature of the surface fermented grains , and well completes the real-time detecting task.

## Introduction

Chinese Baijiu is one of the six major distilled liquors in the world, which has a history of more than two thousand years^1^. It has a unique fermentation and distillation method under solid-state conditions, which is characterized by a long fermentation cycle and rich microorganisms^2^. From grain to Baijiu the technological process includes crushing raw grain, dosing, entering the distillation bucket, distillation, picking liquor, drying, adding Daqu, fermenting, and aging^3^. Liquor refers specifically to Chinese Baijiu in the following text.

Distilling bucket feeding operation is one of the key steps in the distillation process, and it must meet the requirements of "feeding the distilling bucket after steam detection, spread the fermented grains evenly", in order to improve the distillation rate of alcohol in the fermented grains and ensure the production and quality of liquor. "Feeding the distilling bucket after vapor detection" is a method used to detect the overflow of alcohol steam in the distilling bucket. Its main purpose is to heat the surface layer of fermented grains as much as possible while avoiding the "running of vapor" and to obtain a larger temperature difference after spreading the cold material so that the alcohol steam condenses into liquid when cooled, thereby increasing the extraction of alcohol from the fermented grains. In the traditional brewing process, the process of "Feeding the distilling bucket after vapor detection" relies entirely on the brewer's experience. Premature spreading of fermented grains can cause the phenomenon of "liquor steam not reaching the surface of the fermenting grains", while delayed spreading of fermented grains can lead to the phenomenon of "a lot of liquor vapor escaping", both of which can reduce the production of liquor. Therefore, it is of great significance to study the real-time and accurate detection method of fermented grains surface temperature during the feeding process of the distilling bucket for the distillation process of liquor brewing.

Currently, in order to promote the intelligent brewing of liquor, many scholars have conducted in-depth research on the automatic detection of steam during feeding process of the distilling bucket. Li et al.^4^ proposed to use the steam characteristics of the process in the feeding the distilling bucket as the identification target, and utilized image processing to obtain the location of the vaporized area to guide the mechanical structure paving the fermented grains. Yang et al.^5^ used image processing to separate the foreground and background of the fermented grains surface image, and determined the laying area by detecting the liquor vapor overflow feature area in the background. However, both of the above methods are characterized by liquor vapor, which can only be detected after the vapor has risen and cannot be predicted in advance. Tian et al.^6^ and Wang et al.^7^ used an infrared thermal camera to obtain images of fermented grains surface temperature and extracted the image temperature features by combining image pre-processing techniques. Then they proposed methods based on Support Vector Machine (SVM) and BP neural network to detect the vapor. Although this method can predict the location of the vapor in advance through the infrared thermal camera, the SVM and BP methods used were only used for image classification, and could not monitor the surface temperature of the fermented grains and steam changes in real time, which could not satisfy the process requirements of "feeding the distilling bucket after steam detection" in advance.

With the rapid development of deep learning technology, more and more algorithms are being used for target recognition and detection tasks in unstructured environments. The YOLO (You Only Look Once) network is a one-stage detection network that achieves both fast and accurate object detection. This algorithm has been widely used in real-time target detection. However, the algorithm is more difficult to be deployed on low computing power platforms due to cost control and application environment limitations, and the research on lightweighting of the model has gradually attracted attention. Wang, and He^8^ compressed the apple fruitlet detection model before fruit thinning based on YOLO v5s by a channel pruning algorithm, resulting in an average detection time of 8 ms per image and a model size of only 1.4 MB. Li et al.^9^ proposed a real-time tea shoot detection method using YOLO v3 SPP deep learning algorithm with channel and layer pruned, which reduced the number of parameters, inference time, and the model size by 96.82%, 59.62%, and 96.81%, respectively, while the mean average precision was reduced by only 0.40%. Li, Li, Zhao, Su, and Wu^10^ used a lightweight network GhostNet instead of a backbone network and designed a depthwise separable convolution instead of a standard convolution based on the improved YOLO v4 in tea bud detection. Relative to the original YOLOv4, the mean average precision was improved by 1.08%, whereas, the number of parameters and the computational complexity of the proposed model was reduced by 82.36% and 89.11%. A lightweight tea bud detection algorithm based on YOLOv5 was proposed by Gui et al.^11^, incorporating optimizations such as Ghost_conv, BAM, MS-WFF, and CIoU. This method achieved a 9.66% increase in average precision, a 52.402 G and 22.71 M reduction in the floating point operations and the number of parameters, respectively.

In the process of feeding the distilling bucket after vapor detection, the existing methods can only realize the lag detection after the steam overflow, and can not accurately detect the location of the steam, etc. At the same time, in order to effectively reduce the occupancy of the computational resources and improve the deployment performance, an lightweight method YOLO v5ns-DP based on YOLO v5n was proposed. This method can realize the real-time detection of the surface temperature of the fermented grains, and can proactively prejudge the location of the liquor steam in advance to avoid the phenomenon of steam running, so as to improve the production of liquor. The study consists of two main parts as follows: (1) an infrared image dataset of the surface layer of fermented grains was established and YOLO v5n network model was used for training; (2) the model was compressed on the basis of knowledge distillation and channel pruning.

## Materials and methods

### Overview of the method

**Figure 1:**
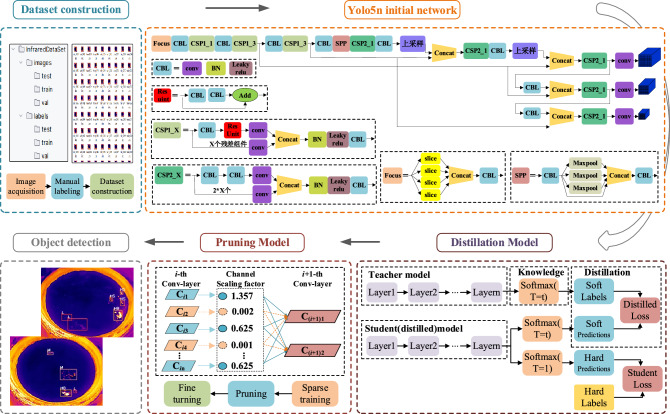
Overall technical route of the proposed the surface temperature detection algorithm.

The overall technical route of this research algorithm is shown in Fig. 1. First, images of the surface layer of fermented grains during the distillation process in the feeding the distilling bucket were acquired using an infrared thermal camera and the target area of the white-hot zone was labeled, so as to establish the temperature detection dataset. Second, YOLO v5n network was used to detect the surface temperature of the fermented grains. Then, using YOLO v5s as the teacher network and YOLO v5n as the student network, a knowledge distillation algorithm was used to migrate the knowledge from the teacher model to the student model to improve the model accuracy. Finally, sparse training and channel pruning were performed to filter and prune non-important channels, and then the pruned network is trained with fine-tuning. Model compression was realized through knowledge distillation and channel pruning while maintaining model accuracy.

### Image acquisition and dataset construction

#### Image acquisition

**Figure 2:**
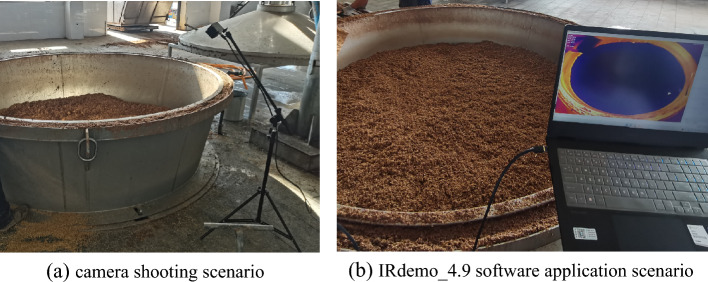
Image acquisition scenarios. (**a**) camera shooting scenario, (**b**) IRdemo_4.9 software application scenario.

Images were acquired at a winery workshop in Shanxi Province, China. Figure 2 shows a scene of image acquisition of the surface of fermented grains during the process of traditional manual feeding distilling bucket. The acquisition equipment consists of a K12E2 in-line infrared thermal camera and the supplementary software IRdemo_4.9. The height of the distilling bucket is 0.9 m and the diameter of the upper rim is 2.2 m. The camera was installed on a bracket 0.6 m from the upper edge of distilling bucket, with the camera optical axis at an angle of 45°-60° to the horizontal plane and aligned with the center of the circle on the upper edge of the distilling bucket, so that the camera can capture the entire fermented grains surface. In this paper, we focus on solving the problem of target detection in pixel space, with special attention to the labeling of upper vapor points. Therefore, a fixed camera mounting position was used in the data acquisition process, ignoring the effect of camera mounting position on the quality of data acquisition.

In addition, image acquisition was performed in July (temperature of 30 ℃) and November (temperature of 15 ℃), respectively. After comparing and analyzing the images, it was found that the infrared camera captured the relative temperature, which could only reflect the temperature difference between the temperature of the upper vapor point and the surrounding temperature, and could not accurately reflect the absolute temperature of the upper vapor point. Since the temperature of the upper vapor point and the surrounding temperature are equally affected by the external ambient temperature, the external ambient temperature has little effect on the quality of the data and the accuracy of the model training.

**Figure 3:**
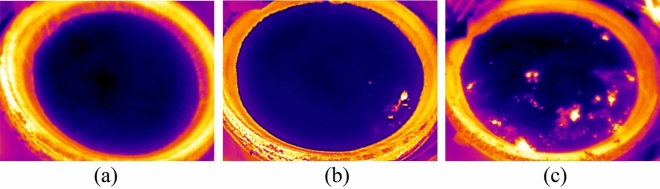
Infrared images of the surface of fermented grains (**a**) Infrared image of the area without white-hot (**b**) Infrared image of localized white-hot areas (**c**) Infrared image of large white-hot areas.

Finally, Fig. 3 shows the collected infrared image with a resolution of 256 pixels × 192 pixels. In the image, the protruding parts such as the distilling bucket body can be clearly seen. Figure 3a shows that the infrared image has a dark blue color with no white-hot area, which indicates that the lower layer of high-temperature liquor vapor has not yet reached the surface of fermented grains. Figure 3b shows an infrared image with white-hot area in some parts of the image, but the majority of the image is in dark blue color, which indicates that the lower layer of high-temperature liquor vapor partially reaches the surface of the fermented grains. Figure 3c shows a large white-hot area in the infrared image, indicating that the liquor vapor reaches the surface of the fermented grains over a large area. After manual selection to remove images with insufficient pixel area and redundancy, 929 infrared images of fermented grains surface were finally selected.

#### Dataset construction

**Figure 4:**
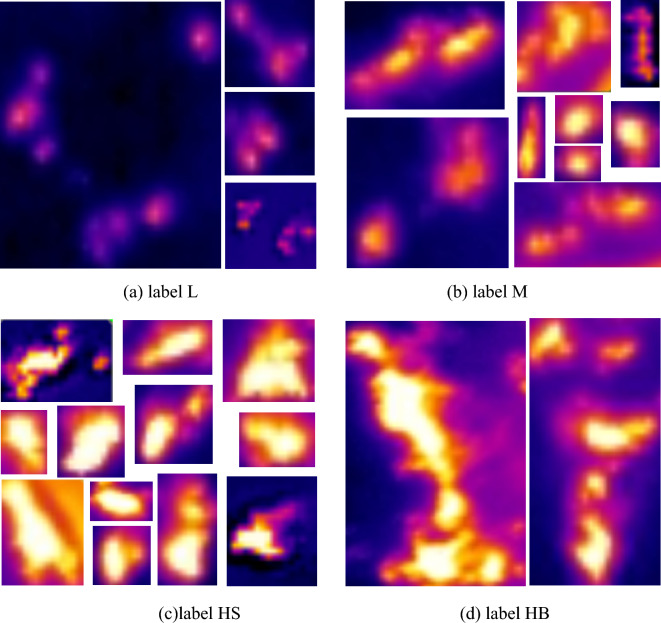
Sample labeling criteria. (**a**) label L, (**b**) label M, (**c**) label HS, (**d**) label HB.

In this study, the white-hot area was used as the target detection region. In order to adapt to the priority principle of feeding trajectory planning at the end of the robotic arm, the samples were divided into four categories, L, M, HS, and HB, according to the brightness and size of the white-hot area of the vapor on the surface layer of the fermented grains during the feeding process. As shown in Fig. 4a, there is a faint brightness in the white-hot region, which is noted as label L. As shown in Fig. 4b, the white-hot area is moderately bright. It indicates that the fermented grains surface has not yet begun to leak and is noted as label M. Figure 4c shows that there is discrete, highlighted, small area of white-hot. This indicates a small area of vapor leakage on fermented grains surface, noted as label HS, which is noted as label HS. As shown in Fig. 4d, there is continuous, highlighted, large white-hot area, which is noted as label HB.

The target areas of the selected image were labeled using the LabelImg, and the labeled box is the smallest outer rectangle of the white-hot area. What’s more, considering that the picture of the waiting for feeding condition does not show a white-hot area and does not require distilling pot feeding operation, the picture of this condition was not labeled. Finally, there were 18 unlabeled images out of 929 infrared images of the fermented grains surface, and the remaining images were labeled with a total of 5360 white-hot areas containing 2633 L labels, 1671 M labels, 1234 HS labels, and 330 HB labels.

**Figure 5:**
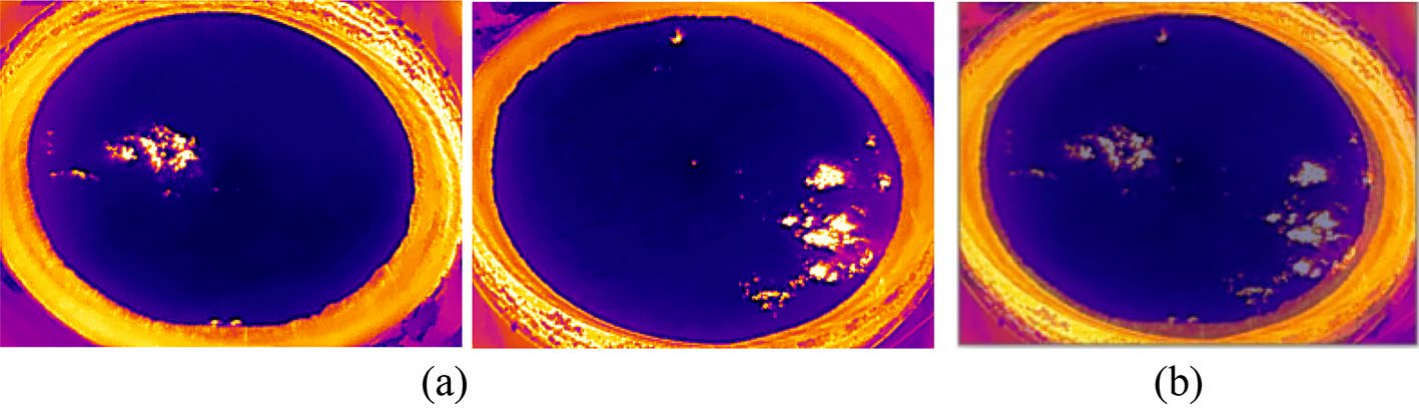
Data-enhanced images based on image processing (**a**) original images (**b**) the resultant image of mixup data enhancement.

In order to improve the richness of the dataset and restore as much as possible the characteristics of the temperature changes on the surface of the fermented grains during the distilling pot feeding process, this study uses several ways to augment the infrared images. These include mixup data enhancement^12^ and random combinations of adding noise, changing brightness, and rotating images to improve the robustness and generalization ability of the model training results. As shown in Fig. 5a is the original image and Fig. 5b is the resultant image by mixup data enhancement method.

All labeled original images were divided into training, validation and test sets in the ratio of 70%:15%:15%. The training set samples were extended by image enhancement and labeled using LabelImg. Finally, the final augmented dataset is listed in Table 1.

**Table 1:** Infrared image dataset of fermented grains surface.

DataSet	Original training set	Post-enrichment training set	Valid set	Test set	Number of infrared images	Image size
InfraredDataSet	721 images	1442 images	104 images	104 images	1650 images	256 pixels × 192 pixels

### Fermented grains surface temperature detection based on YOLO v5n

In recent years, deep-learning-based image detection networks have been divided into two-stage and one-stage detection networks^13^. Faster R-CNN^14^ is a classical two-stage detection network with high detection accuracy, but the inference speed is relatively slow and cannot meet the real-time requirements. YOLO, SSD, and RetinaNet are representative single-stage detection networks, which are usually faster than Faster R-CNN in terms of speed and suitable for application scenarios requiring real-time performance. Under resource constraints, SSD^15^ and RetinaNet^16^ may have a slight disadvantage in processing speed compared to YOLO. YOLO models are characterized by simplicity and are easier to train and deploy. In addition, YOLO has achieved a better balance between accuracy and speed, which is particularly suitable for application scenarios that require rapid deployment and real-time target detection.

YOLO v5 series models have better versatility, ease of use, and compatibility, and balances recognition accuracy with detection speed^17–20^. Among them, the YOLO v5n version is the less structured and more accurate target detection model in the series. Compared with other models in the YOLO v5 series, YOLO v5n greatly reduces the number of model parameters and computation while ensuring a certain recognition accuracy^21^. Therefore, it is suitable for target detection tasks with fewer categories and simple features. In this study, the YOLO v5n model was used for training as the original model. The hyperparameters for model training are listed in Table 2.

**Table 2:** Hyperparameters for model training.

Batch size	Initial learning rate	Momentum	Weight decay factor	IOU threshold	Number of iterations
4	0.01	0.937	0.0005	0.5	1000 epochs

### Fermented grains surface temperature detection based on knowledge distillation

In this study, YOLO v5s was selected as the teacher network and YOLO v5n as the student network to improve the accuracy of the student network in the task of detecting the surface temperature of the fermented grains through knowledge distillation for later pruning.

Knowledge distillation training is different from traditional model training. Softmax is commonly used as the output layer in traditional models to generate probabilities for different categories. When the probability distribution entropy generated by Softmax output is relatively low, the values for negative labels tend to approach 0. As a result, the contribution of negative labels to the loss function becomes negligible. However, negative labels also contain a great deal of information. Some negative labels may correspond to higher probabilities than others, and even contain more information than positive labels. In order to obtain more information from negative labels, knowledge distillation introduces a temperature variable, denoted by *T*. By adjusting the value of *T*, the entropy of the Softmax output probability distribution can be increased, thus amplifying the information carried by negative labels.

1 $$ q_{i} = \frac{{\exp (z_{i} /T)}}{{\sum\limits_{j} {\exp (z_{j} /T)} }} $$

where *q*_*i*_ is the "softened" probability vector, obtained by exponential operation and normalization; Z_*i*_ indicates the logit value for the current category; *j* denotes the number of output nodes (number of categories); Z_*j*_ represents the logit value for each category output by the full connectivity layer; *T* is the temperature parameter, and when *T* = 1, the function is the original Softmax function. The knowledge distillation process was shown in the knowledge distillation model in Fig. 1^22,23^. First, the teacher network model is trained and the logits output of the teacher network is divided by the *T* after doing Softmax calculation to get the soft label value. Then, the same training as for the teacher network is performed to get the logits output. Next, a two-step calculation is performed. The first step is to perform a Softmax calculation by dividing the logits output of the student network by the same *T* as the teacher model to obtain the soft prediction. Soft predictions were compared to soft labels, and the difference between the two probability distributions was measured using the distillation loss function. The second step is to perform Softmax computation on the logits output of the student network to get the hard predicted values. The hard predicted values were compared to the actual labels and the difference between them was measured using the student loss function. The two parts of the loss function are added together to get the total loss function, which is calculated as

2 $$ {\text{V}}_{loss} = \left( {{1} - \alpha } \right){\text{V}}_{loss - SL} + \alpha T^{{2}} {\text{V}}_{loss - KD} $$

where V_*loss*_ is the value of total loss function; V_*loss-SL*_ is the value of the student loss function; V_*loss-KD*_ is the value of distillation loss function; *α* is the scaling factor, which is used to adjust the weights of the two loss functions. When *α* is equal to zero, this corresponds to the network not being distilled and trained using only the student loss function.

The same training strategy as the original YOLO v5n model was used for the training process in this study. The temperature parameter was taken as *T* = 20 and the scaling factor was taken as *α* = 0.5.

### Fermented grains surface temperature detection based on knowledge distillation channel pruning

The YOLO v5n model in this study can accurately detect surface temperature of fermented grains, but the structure and the number of parameters of the model have not yet reached the optimal effect, which occupies more computational resources. In order to improve the usefulness of the model and reduce the computational effort, the model is compressed using channel pruning^24,25^.

**Figure 6:**
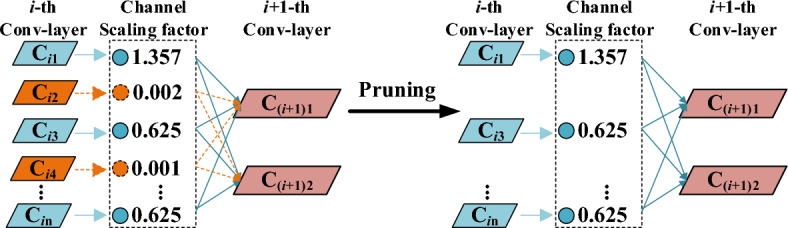
Principles of channel pruning.

The channel pruning process is shown in Fig. 6. First, Sparse training using L_1_ norm on the scaling factor of the BN layer in the model to find the channel where the scaling factor tends to zero^26^. The closer scaling factor is to 0, the less important the corresponding output is to the final result^27^. After sparse training, the unimportant channels were pruned to obtain a smaller pruned model. Finally, fine tuning the pruned model to overcome the average accuracy decrease caused by the channel pruning algorithm.

The formulas for the BN layer scaling factor evaluation method are shown in (3) and (4):

3 $$ \hat{x}_{i} = \frac{{x_{i} - \mu_{B} }}{{\sqrt {\sigma_{B}^{2} + \varepsilon } }} $$

4 $$ y_{i} = \gamma \hat{x}_{i} + \beta $$

where *x*_*i*_ and *y*_*i*_ are the inputs and outputs of the BN layer; *μ*_B_ and *σ*_B_^2^ are the mean and variance of the batch data; *ε*denote the tiny positive numbers used to avoid divisors of 0; *γ* and *β* are the BN layer scaling factor and bias, respectively, with smaller scaling factors usually corresponding to less important channels.

An L1-norm is done on the BN layer scaling factor (as shown in Eq. (3)) to force learning to sparse *γ*. A global ranking is done by the magnitude of the absolute value of *γ* to evaluate the importance of the channels. Then, the pruning ratio is set to prune unimportant channels.

5 $$ L = \sum\limits_{{\left( {x,y} \right)}} {l\left[ {f\left( {x,W} \right),y} \right]} + \lambda \sum\limits_{\gamma \in \Gamma } {g(\gamma )} $$

where the first summation term is the normal training loss function, (*x, y*) denotes the input and target of training, *W* denotes the trainable weights, and *l*( ) denotes the normal training loss; The second summation term denotes sparse training by L1-norm on the scaling factor, g(*γ*) denotes the penalty function for the scaling factor, Γdenotes the scaling layer parameter, *λ* denotes the balance factor between normal and sparse training, i.e., the sparsity rate.

The sparse process requires a balance between accuracy and sparsity, which is achieved by adjusting the sparsity rate. Larger coefficients cause the *γ* tend to zero more quickly, but the average recognition accuracy decreases; smaller coefficients cause a slower tendency to zero, but a more stable accuracy. After sparse training, the model is compressed by removing unimportant channels in the convolution layer based on the mean ordering of the scaling factors of the BN layers. The accuracy and memory usage are considered together so as to determine the number of deleted channels to realize pruning. Once the model has been pruned, there is a notable reduction in the number of parameters and model size. However, this reduction often leads to a significant decrease in model accuracy. In order to overcome the problem of excessive loss of model accuracy after pruning, it is necessary to fine-tune the model after pruning. In this study, fine-tuning training was performed by loading the pruning weights file and setting the number of training iterations to 1000 epochs.

## Experiments and result analysis

### Training environment and evaluation indicators

#### Training environment

All training in this study was performed under the Win10 operating system with Intel(R) Core (TM) i7-7700 CPU@3.60 GHz processor and NVIDIA GeForce GTX 950 graphics board. We used PyTorch1.10, PyCharm and Python3.8.5. Meanwhile, all comparison algorithms were run in the same environment to ensure the comparability of the experiments.

#### Evaluation criteria

In order to validate the model performance, model recognition performance metrics, computational performance metrics, and model memory footprint were selected to evaluate the model in this study. The mean average precision (mAP) was used to measure recognition performance, which was calculated using IOU threshold of 0.5. Parameters and floating-point operations (FLOPs) were used to measure computational performance. In addition, model size and FPS were considered. These metrics provide insight into accuracy, efficiency, and resource requirements, providing guidance for future model improvements.

#### Results based on knowledge distillation

To compensate for the inevitable decrease in accuracy of the model during subsequent pruning, this study uses a knowledge distillation algorithm to migrate the knowledge from the YOLO v5s teacher model to the YOLO v5n student model. The distilled model was named YOLO v5ns-D. As clearly shown in Table 3, the YOLO v5ns-D model improves the mAP value by 2.9 compared with the YOLO v5n model, while the model size, number of parameters, FLOPs and FPS are basically unchanged. The result showed that the knowledge distillation was successful in improving the model performance and laid the foundation for the subsequent pruning process.

**Table3:** Performance Comparison of YOLO v5s, YOLO v5n, and YOLO v5ns-D Models.

Model	*mAP*(0.5) /%	FPS/fps	Model size/MB	Number of parameters /M	FLOPs/G
YOLO v5s	84.8	63.285	13.6	7.030	0.958
YOLO v5n	81.1	81.665	3.56	1.769	0.254
YOLO v5ns-D	84	81.927	3.56	1.769	0.254

### Results based on channel pruning

#### Results of sparse training

In order to maintain a good recognition performance while sparse training, the choice of the sparsity rate is very important, which determines the proportion of non-zero parameters to be retained in the model. Experimentation and validation are required to determine the most appropriate sparsity rate coefficient.

**Figure 7:**
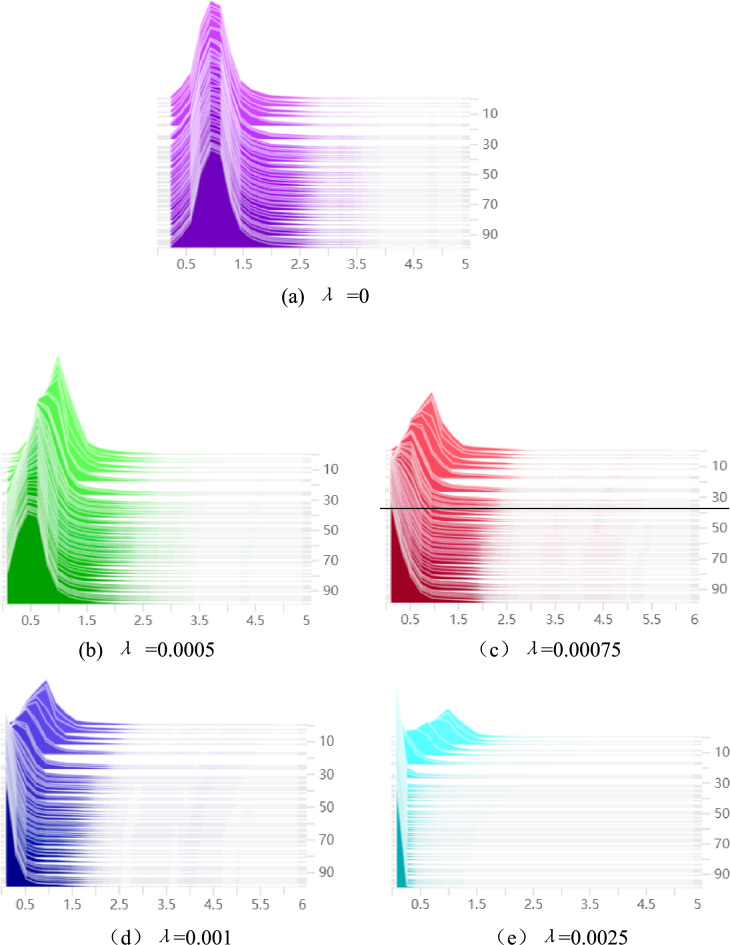
BN layer γ coefficient under different sparsity rates (λ). (**a**) λ = 0, (**b**) λ = 0.0005, (**c**) λ = 0.00075, (**d**) λ = 0.001, (d) λ = 0.0025.

When different sparsity rates are to be set, the model's BN layer weights and mean average precision change accordingly. The BN layer γ coefficient distribution of the original detection model is shown in Fig. 7a, which was nearly normally distributed overall. Figure 7b–e show the BN layer γ coefficient distribution for sparsity rates of 0.0005,0.00075,0.001, 0.0025, respectively, and the number of sparse training epochs is 100. It can be seen that the distribution centers of γ coefficients of all BN layers gradually moved closer to 0 with training, and the larger the sparsity rate is, the faster the distribution centers of γ coefficients moved closer to 0 and the more concentrated the distribution is. After comparison, six datasets were chosen to be taken at 0.00005 intervals between sparsity rates from 0.00075 to 0.001 to analyze the model performance. The results of mean average precision at different sparsity rates is shown in Table 4. It can be seen that the mAP is highest when the sparsity rate is 0.0009, so the sparsity rate is selected as 0.0009 in this study.

**Table 4:** Model performance at different sparsity rates (*λ*).

sparsity rates	0.00075	0.0008	0.00085	0.0009	0.00095	0.001
mAP/%	73.4	74.6	73.9	75.2	71.6	72.6

#### Results of pruning and fine-tuning

**Figure 8:**
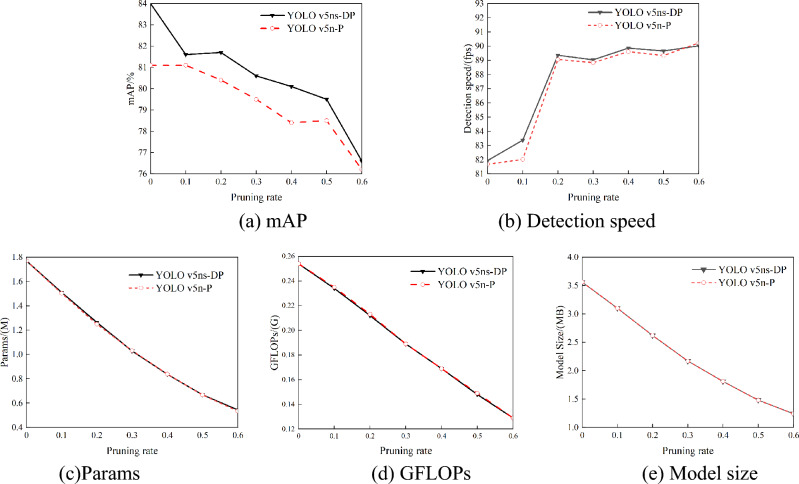
Model performance under different pruning rates. (**a**) mAP, (**b**) Detection speed, (**c**)Params, (**d**) GFLOPs, (**e**) Model size.

After determining the sparsity rate, in this study, YOLO v5ns-D model and the original model YOLO v5n are pruned with different proportions and fine-tuned, and the fine-tuned models were named YOLO v5ns-DP and YOLO v5n-P. The optimal pruning rate is selected by comparative analysis of model performance. When the pruning rate is greater than 0.681, one of the channels in the convolution layer will be pruned as a whole, affecting the model structure and causing a significant reduction in model accuracy. To determine the optimal channel pruning coefficient, different pruning coefficients were tested in the experiments, using a step size of 0.1. The results of the experiments can be seen in Fig. 8.

As clearly shown in Fig. 6, the number of parameters, GFLOPs and model size of YOLO v5ns-DP and YOLO v5n-P models decreased to different degrees with the increasing of pruning rate. When the pruning rate is 0.2, the mAP of the YOLO v5ns-DP model is 81.7, which is 0.6 higher than the mAP of the original YOLO v5n model, whereas the number of parameters, the computation and the model size are decreased by 28.6%, 16.5% and 26.4%, respectively. Meanwhile, the distillation and then pruning model improved the mAP values for the same pruning rate compared with the direct pruning of the original model, while the number of model parameters, GFLOPs and the size of the model were basically unchanged. With a pruning rate of 0.2, for example, the mAP value of YOLO v5ns-DP is 1.5 higher than that of YOLO v5n-P. It is demonstrated that knowledge distillation and then pruning can effectively improve the model performance and avoid information loss and performance loss caused by relying only on pruning fine-tuning training.

**Figure 9:**
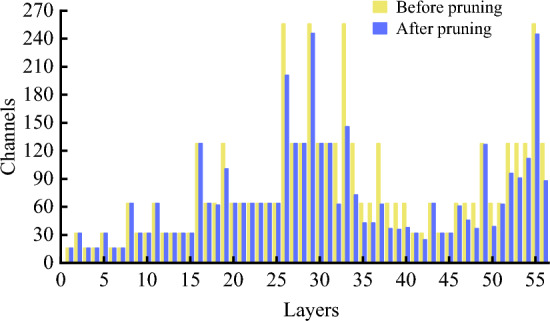
Channel changes before and after pruning.

The change in the number of the channel in each convolution layer of the model after pruning at 0.2 pruning rate is shown in Fig. 9. It can be seen that the number of channels in most convolution layers is effectively reduced, with an average of about 13 channels per layer being pruned, indicating that the pruning algorithm is effective.

### Comparative experiments and analysis of results

To evaluate the effectiveness of the proposed method, this study compares our proposed YOLO v5ns-DP model with YOLO v5n, Faster R-CNN and SSD. An enhanced dataset of infrared images of fermented grains surface layers was used to train the detection models for the four algorithms, and then a test set was used to evaluate the performance of the different detection algorithms. The detailed results of the experiment are shown in Table 5.

**Table 5:** Performance Comparison of different Models.

Model	*mAP*(0.5)/%	FPS/fps	Model size/MB	Number of parameters/M
Faster R-CNN	82.4	21.853	68.1	20.358
SSD	81.3	51.962	40.3	14.236
YOLO v5n	81.1	81.665	3.56	1.769
YOLO v5ns-DP	81.7	89.891	2.62	1.263

The above experimental results show the significant advantages of the YOLO v5ns-DP in target detection tasks. Compared with the classical two-stage detection network Faster R-CNN, although the mAP was reduced by 0.7, the model size and the number of parameters were reduced by 96.1% and 93.8%, respectively, while the detection speed was improved by a factor of three. Also as a single-stage detection network, the model size and the number of parameters of YOLO v5ns-DP were reduced by 89.8% and 91.1%, respectively, and the detection speed was improved by 72.9% compared to SSD, although there was no significant difference in mAP. In addition, compared with YOLO v5n, although the number of parameters and model size were reduced by 28.6% and 26.4%, respectively, the mAP was improved by 0.6 and the detection speed reached 89.891 FPS. These results show that YOLO v5ns-DP has a smaller model size and is easier to deploy on low-computing-power devices while balancing detection speed and accuracy, which reduces cost and improves efficiency.

**Figure 10:**
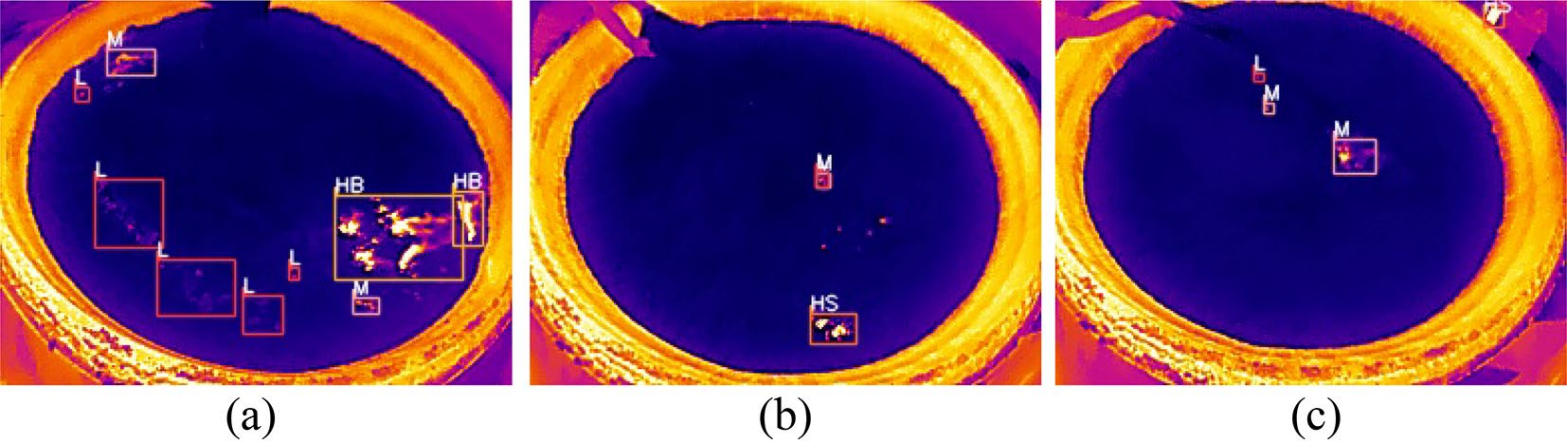
Detection effect images of YOLO v5ns-DP model.

Figure 10 shows the effectiveness of the YOLO v5ns-DP in detecting temperature changes in the surface layer of the fermented grains. In Fig. 10a, five L-targets, two M-targets and two HB-targets were successfully identified and could be automatically labeled. In Fig. 10b, one M-target and one HS-target were successfully recognized, but there was a white-hot region leakage. In Fig. 10c, one L-target and two M-targets were successfully recognized, but at the same time there were cases where white-hot targets were detected outside the distilling pot region.

In the overall analysis, YOLO v5ns-DP showed good performance in detecting the temperature change of the surface layer of fermented grains, with high detection accuracy and target recognition ability. However, the model still has some room for improvement, such as the leakage problem and the false detection problem which need to be further optimized. In the future work, we will further study how to improve the accuracy of the algorithm in the practice of target detection of temperature changes in the surface layer of fermented grains.

## Conclusion

In this study, a lightweight method of fermented grains surface temperature detection was proposed on the basis of the YOLO v5n network. The knowledge from the YOLO v5s teacher model was migrated to the YOLO v5n student model through the knowledge distillation algorithm. Then, the knowledge distilled detection model was compressed by sparse training, channel pruning, and fine-tuning. Finally, the compressed model was named YOLO v5ns-DP. After compression, the lightweight model YOLO v5ns-DP improved the mAP value by 0.6 and achieved a detection speed of 89.891 FPS compared with the original model YOLO v5n, while the number of parameters, GFLOPs and model size reduced by 28.6%, 16.5% and 26.4%, respectively. All the results demonstrate that it is practical to use this method to achieve both rapid and accurate detection of fermented grains surface temperature in the process of feeding the distilling bucket. At the same time, the lightweight model not only provided a theoretical basis for the model to be deployed in the edge computing device, but also provided a technical basis for the intelligence of “feeding the distillation bucket after vapor detection”.

## Data Availability

Data or code presented in this study is available. The computer code that support the findings of this study have been deposited on Zenodo with the primary accession link: 10.5281/zenodo.11165115.
